# The necessity of implementing the World Health Organization Checklist for trauma patients in Emergency Departments throughout the country

**Published:** 2019-08

**Authors:** Alireza Ahmadi

**Affiliations:** ^*a*^Department of Anesthesiology, Critical Care and Pain Management, Kermanshah University of Medical Sciences, Kermanshah, Iran.

## Abstract

Injury is one of the main causes of mortality and disability in the world. Improving the trauma care process is necessary to reduce this burden in countries at all levels of the economy. One of the important components of improving the care process in each field is to reduce medical errors. Using a checklist in other medical areas has been effective in improving care and reducing medical mistakes. Field observations indicate that almost all of the emergency departments in hospitals and trauma centers in the country do not use this check list. In this short essay, efforts have been made to emphasize the necessity for coercion with the necessary training to use this checklist from the Department of Health's treatment deputy.

**Keywords:**

Trauma patients, World Health Organization, Emergency, Checklist

